# Association of relapse-linked *ARID5B* single nucleotide polymorphisms with drug resistance in B-cell precursor acute lymphoblastic leukemia cell lines

**DOI:** 10.1186/s12935-020-01524-0

**Published:** 2020-09-04

**Authors:** Minori Tamai, Meixian Huang, Keiko Kagami, Masako Abe, Shinpei Somazu, Tamao Shinohara, Daisuke Harama, Atsushi Watanabe, Koshi Akahane, Kumiko Goi, Kanji Sugita, Hiroaki Goto, Masayoshi Minegishi, Shotaro Iwamoto, Takeshi Inukai

**Affiliations:** 1 Department of Pediatrics, School of Medicine, University of Yamanashi, Shimokato, Chuo, Yamanashi 1110 Japan; 2 Yamanashi Red Cross Blood Center, Kofu, Japan; 3 Division of Hematology/Oncology, Kanagawa Children’s Medical Center, Yokohama, Japan; 4 Tohoku Block Center, Japanese Red Cross Society, Sendai, Japan; 5 Department of Pediatrics, Mie University Graduate School of Medicine, Tsu, Japan

**Keywords:** ARID5B, B-cell precursor acute lymphoblastic leukemia, Drug sensitivities, Single nucleotide polymorphism

## Abstract

**Background:**

The genetic variants of the *ARID5B* gene have recently been reported to be associated with disease susceptibility and treatment outcome in childhood acute lymphoblastic leukemia (ALL). However, few studies have explored the association of ARID5B with sensitivities to chemotherapeutic agents.

**Methods:**

We genotyped susceptibility-linked rs7923074 and rs10821936 as well as relapse-linked rs4948488, rs2893881, and rs6479778 of *ARDI5B* by direct sequencing of polymerase chain reaction (PCR) products in 72 B-cell precursor-ALL (BCP-ALL) cell lines established from Japanese patients. We also quantified their *ARID5B* expression levels by real-time reverse transcription PCR, and determined their 50% inhibitory concentration (IC50) values by alamarBlue assays in nine representative chemotherapeutic agents used for ALL treatment.

**Results:**

No significant associations were observed in genotypes of the susceptibility-linked single nucleotide polymorphisms (SNPs) and the relapsed-linked SNPs with *ARID5B* gene expression levels. Of note, IC50 values of vincristine (VCR) (median IC50: 39.6 ng/ml) in 12 cell lines with homozygous genotype of risk allele (C) in the relapse-linked rs4948488 were significantly higher (p = 0.031 in Mann–Whitney U test) than those (1.04 ng/ml) in 60 cell lines with heterozygous or homozygous genotypes of the non-risk allele (T). Furthermore, the IC50 values of mafosfamide [Maf; active metabolite of cyclophosphamide (CY)] and cytarabine (AraC) tended to be associated with the genotype of rs4948488. Similar associations were observed in genotypes of the relapse-linked rs2893881 and rs6479778, but not in those of the susceptibility-linked rs7923074 and rs10821936. In addition, the IC50 values of methotrexate (MTX) were significantly higher (p = 0.023) in 36 cell lines with lower *ARID5B* gene expression (median IC50: 37.1 ng/ml) than those in the other 36 cell lines with higher expression (16.9 ng/ml).

**Conclusion:**

These observations in 72 BCP-ALL cell lines suggested that the risk allele of the relapse-linked SNPs of *ARID5B* may be involved in a higher relapse rate because of resistance to chemotherapeutic agents such as VCR, CY, and AraC. In addition, lower *ARID5B* gene expression may be associated with MTX resistance.

## Background

**Fig. 1:**
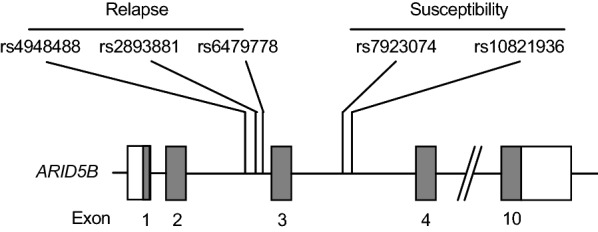
Locations of relapse- and susceptibility-linked SNPs of *ARID5B*

B-cell precursor acute lymphoblastic leukemia (BCP-ALL) is the most common neoplasm in children. Recent genome-wide association studies (GWAS) on pediatric patients with BCP-ALL have identified common single nucleotide polymorphisms (SNPs) associated with disease susceptibility [1–6]. SNPs located in intron 3 of the *ARID5B* gene (i.e. rs7923074 and rs10821936; Fig. 1) are the most significant and recapitulated SNPs in various races, including Asian populations [7–10]. *ARID5B* belongs to the AT-rich interactive domain (ARID) family and acts as a transcription coactivator that binds to the 5′-AATA[CT]-3′ core sequence [11, 12]. Although the direct mechanism for leukemogenesis is not fully understood, the risk allele of susceptibility-linked SNPs in intron 3 of the *ARID5B* gene may alter the transcription network involved in normal lymphopoiesis by disrupting *ARID5B* expression [13]. Interestingly, further GWAS on pediatric ALL patients revealed that the other SNPs located in intron 2 of the *ARID5B* gene (i.e. rs4948488, rs2893881, and rs6479778; Fig. 1) were significantly associated with their relapse rate [14]. This clinical observation suggests that the genotype of these relapse-linked SNPs of *ARID5B* may be associated with the responses to chemotherapeutic agents. Nevertheless, few studies have focused on the association of ARID5B with drug sensitivities in BCP-ALL [15].

Therefore, to address this issue, we analyzed any association of *ARID5B* genotype with *ARID5B* gene expression and drug sensitivity in a series of BCP-ALL cell lines. We found that genotypes of the relapse-linked SNPs of *ARID5B* are associated with resistance to several chemotherapeutic agents.

## Materials and methods

### Cell lines

We used 72 BCP-ALL cell lines that were established from Japanese patients as described in detail previously [16] (Additional files 1, 2: Tables S1, S2). Among the 72 cell lines, 15 cell lines were *MEF2D* fusion-positive, 14 cell lines were *BCR/ABL1*-positive, 13 cell lines were *TCF3/PBX1*-positive, 12 cell lines were *MLL* (*KMT2A*)-rearranged, 4 cell lines were *ETV6/RUNX1*-positive, 3 cell lines were *TCF3/HLF*-positive, and 2 cell lines were *BCR/ABL1*-like. No hyperdiploid cell lines were included. Forty-six cell lines were sequentially established in our laboratory from 1980 to 2011, while 24 cell lines were provided by 10 institutes. Two additional cell lines were purchased from American Type Culture Collection (ATCC). All cell lines were maintained in RPMI1640 media with 10% fetal calf serum (FCS) at 37 °C under a 21% O_2_ and 5% CO_2_ atmosphere.

### Real-time reverse transcription polymerase chain reaction (RT-PCR)

Total RNA was extracted from each cell line using TRIzol reagent (Invitrogen, Carlsbad, CA), and reverse transcription reactions were performed using a random hexamer (Amersham Bioscience, Buckinghamshire, UK) and Superscript II Reverse Transcriptase (Invitrogen). To remove unreacted mRNA, the samples were treated with RNase (Invitrogen) after the reaction. Real-time reverse transcription polymerase chain reaction (RT-PCR) analyses of *ARID5B* were performed using a TaqMan probe kit (Hs01382781_m1). Gene expression level of the beta-actin (*ACTB)* gene was also examined as an internal control using a TaqMan probe kit (Hs01060665_g1).

### SNP genotyping

Genomic DNA was extracted from each cell line using a PureLink Genomic DNA Mini Kit (Invitrogen). Genomic regions containing two representative susceptibility-linked SNPs (rs7923074 and rs10821936) and three representative relapse-linked SNPs (rs4948488, rs2893881 and rs6479778) of *ARID5B* in 72 BCP-ALL cell lines were amplified using primers described in Table 1. Then, genotypes of five SNPs in each cell line were determined after direct sequencing of each genomic PCR product using forward primers for rs7923074, rs4948488, and rs2893881 as well as a reverse primer for rs10821936 and rs6479778.

**Table 1:** Primers for SNP genotyping

SNP	Forward primer	Reverse primer	Product size (bp)
rs4948488	5′-GAGCATAACACTGGAATTGGGC-3′	5′-AACTCCTTTCAGGTTGCCAT-3′	107
rs2893881	5′-TGTAATGGGGAGAACAGTTGGG-3′	5′-ATGTACCACCTCGAAGCCTG-3′	113
rs6479776	5′-TGGGATGTTCAGGGAAGACTG-3′	5′-TCACCTAGCATCCCAAGGAC-3′	121
rs7923074	5′-TGTCTCTCCCTGACTGGACC-3′	5′-GCACACAGAAGGGGCTAGAG-3′	235
rs10821936	5′-TTTATGCTGCCGCTAATGCC-3′	5′-GGGACTAACCATTAGTATCCCCC-3′	155

*SNP* single nucleotide polymorphism

### AlamarBlue assay

Fifty percent inhibitory concentration (IC50) values of prednisolone (Pred), dexamethasone (Dex), vincristine (VCR), daunorubicin (DNR), L-asparaginase (L-Asp), cytarabine (AraC), methotrexate (MTX), mercaptopurine (6MP), and mafosfamide [Maf; active metabolite of cyclophosphamide (CY)] were determined using the alamarBlue cell viability assay (Bio-Rad Laboratories, Hercules, CA) as previously reported [17]. Cells (1–4 × 10^5^) were placed onto 96-well flat bottom plates in the presence or absence of seven separate concentrations of each drug in triplicate. The cells were cultured for 44 h to determine the DNR, VCR and CY (Maf) sensitivities and for 68 h to determine Pred, Dex, L‐Asp, MTX, and 6MP; 20 µL of alamarBlue was then added. After incubation for an additional 6 h in the presence of alamarBlue, the optimal density was read on a spectrophotometer at 570 nm using 600 nm as a reference wavelength. Cell viability was calculated by the ratio of the optical density of the treated wells to that of the untreated wells as a percentage. The concentration of each agent required to reduce the viability of the treated cells to 50% of the untreated cells (IC50 value) was calculated and the median IC50 value of three independent assays was determined.

### Cell cycle analysis

Each cell line at a density of 0.5 × 10^5^ cells/ml was cultured with fresh RPMI1640 media with 10% FCS for 24 h. Then, cell cycle analysis was performed using flow cytometry after PI staining as previously reported [18]. The median percentage of G0/G1 dormancy phase was determined in three independent analyses.

### Statistics

We applied Fisher’s exact test for comparison of allele frequencies between cell lines and Japanese population in HapMap project database (https://www.ncbi.nlm.nih.gov/variation/news/NCBI_retiring_HapMap/). Mann–Whitney U test was always applied for comparisons between two groups of cell lines using R (version 3.5.1) statistical software.

## Results

### Genotype of susceptibility-linked and relapse-linked SNPs of *ARID5B* in BCP-ALL cell lines

We first analyzed *ARID5B* genotypes in 72 BCP-ALL cell lines established from Japanese patients [16]. Our cell line bank contained 15 MEF2D fusion-positive, 14 *BCR/ABL1*-positive, 13 *TCF3/PBX1*-positive, 12 *MLL* (*KMT2A*)-rearranged, 4 *ETV6/RUNX1*-positive, 3 *TCF3/HLF*-positive, and 2 *BCR/ABL1*-like cell lines, but no hyperdiploid cell lines (Additional files 1, 2: Table S1, S2). Thus, the majority of our cell lines had been established from BCP-ALL with high or intermediate risk karyotypes. Among 65 cell lines with basic records of cell line establishment, 28 and 37 cell lines were established from the samples at diagnosis and those at relapse, respectively (Additional files 1, 2: Table S1, S2). We determined genotypes of two representative susceptibility-linked SNPs [14] (rs7923074 and rs10821936, Fig. 1) and three representative relapse-linked SNPs [14] (rs4948488, rs2893881, and rs6479778, Fig. 1) in each cell line after direct sequencing of each genomic PCR product. Allele frequencies of each SNP in BCP-ALL cell lines were in Hardy–Weinberg equilibrium. Due to linkage disequilibrium, genotypes of rs7923074 and rs10821936 were identical in 71 of 72 cell lines. Genotypes of rs2893881 and rs6479778 were also identical in 71 cell lines. In the HapMap project database (Table 2), we compared the allele frequency of each SNP between our cell lines and the Japanese population, but no significant differences were observed in the genotypes of both the susceptibility-linked SNPs and the relapse-linked SNPs of *ARID5B*. We also compared the allele frequency of each SNP between 28 cell lines established at diagnosis and 37 cell lines established at relapse, but no significant differences were observed (data not shown).

**Table 2:** Comparison of SNP allele frequencies between BCP-ALL cell lines and the Japanese population

Chr position	SNP	Risk allele	72 BCP-ALL cell lines					HapMap JPT					OR (95%CI)	P value
			Genotype			Allelic frequency		Genotype			Allelic frequency			
Susceptibility-linked														
63,723,440	rs7923074	A	A/A	A/C	C/C	A	C	A/A	A/C	C/C	A	C	0.83	0.37
			(n = 20)	(n = 28)	(n = 24)	(n = 68)	(n = 76)	(n = 38)	(n = 102)	(n = 32)	(n = 178)	(n = 166)	(0.55–1.26)	
			0.278	0.389	0.333	0.472	0.528	0.221	0.593	0.186	0.517	0.483		
63,724,773	rs10821938	A	A/A	A/C	C/C	A	C	A/A	A/C	C/C	A	C	0.86	0.49
			(n = 20)	(n = 29)	(n = 23)	(n = 69)	(n = 75)	(n = 38)	(n = 102)	(n = 32)	(n = 178)	(n = 166)	(0.57–1.29)	
			0.278	0.403	0.319	0.479	0.521	0.221	0.593	0.186	0.517	0.483		
Relapse-linked														
61,925,395	rs4948488	C	T/T	T/C	C/C	T	C	T/T	T/C	C/C	T	C	1.41	0.09
			(n = 30)	(n = 30)	(n = 12)	(n = 90)	(n = 54)	(n = 52)	(n = 82)	(n = 38)	(n = 186)	(n = 158)	(0.93–2.16)	
			0.417	0.417	0.167	0.625	0.375	0.302	0.477	0.221	0.541	0.459		
61,928,913	rs2893881	C	T/T	T/C	C/C	T	C	T/T	T/C	C/C	T	C	1.29	0.26
			(n = 34)	(n = 28)	(n = 10)	(n = 96)	(n = 48)	(n = 62)	(n = 80)	(n = 26)	(n = 204)	(n = 132)	(0.84–2.00)	
			0.472	0.389	0.139	0.667	0.333	0.369	0.476	0.155	0.611	0.389		
61,929,318	rs6479778	T	C/C	C/T	T/T	C	T	C/C	C/T	T/T	C	T	1.28	0.26
			(n = 35)	(n = 27)	(n = 10)	(n = 97)	(n = 47)	(n = 66)	(n = 80)	(n = 26)	(n = 212)	(n = 132)	(0.84–1.97)	
			0.486	0.375	0.139	0.674	0.326	0.384	0.465	0.151	0.616	0.384		

*BCP-ALL* B-cell precursor acute lymphoblastic leukemia, *Chr* chromosome, *SNP* single nucleotide polymorphism, *OR* odds ratio, *95% CI* 95% confidence interval

### No association of susceptibility or relapse-linked SNPs of *ARID5B* with *ARID5B* expression

**Fig. 2:**
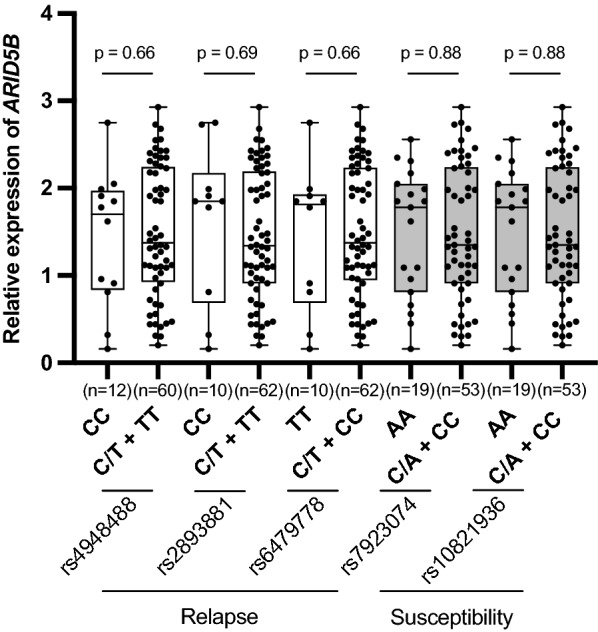
Association of relapse- and susceptibility-linked SNP genotypes with *ARID5B* gene expression. Relative *ARID5B* gene expression levels were compared between cell lines with homozygous genotype of risk allele and those with heterozygous or homozygous genotypes of non-risk allele in each SNP. P-value in Mann–Whitney U test is indicated at the top of each SNP

Since both the susceptibility-linked SNPs and the relapse-linked SNPs of *ARID5B* are located in intronic regions, we next performed an expression quantitative trait locus (eQTL) analysis. We quantified the *ARID5B* gene expression level in each cell line by real-time RT-PCR using *ACTB* gene expression as an internal control. However, in the eQTL analysis of 72 BCP-ALL cell lines, neither genotypes of susceptibility-linked rs7923074 and rs10821936 nor those of relapse-linked rs4948488, rs2893881, and rs6479778 were significantly associated with *ARID5B* expression level (Fig. 2). These observations demonstrated that genotypes of both susceptibility-linked SNPs and relapse-linked SNPs of *ARID5B* were not clearly associated with *ARID5B* expression levels in the BCP-ALL cell lines.

### Association of relapse-linked SNPs of *ARID5B* with drug sensitivity

**Fig. 3:**
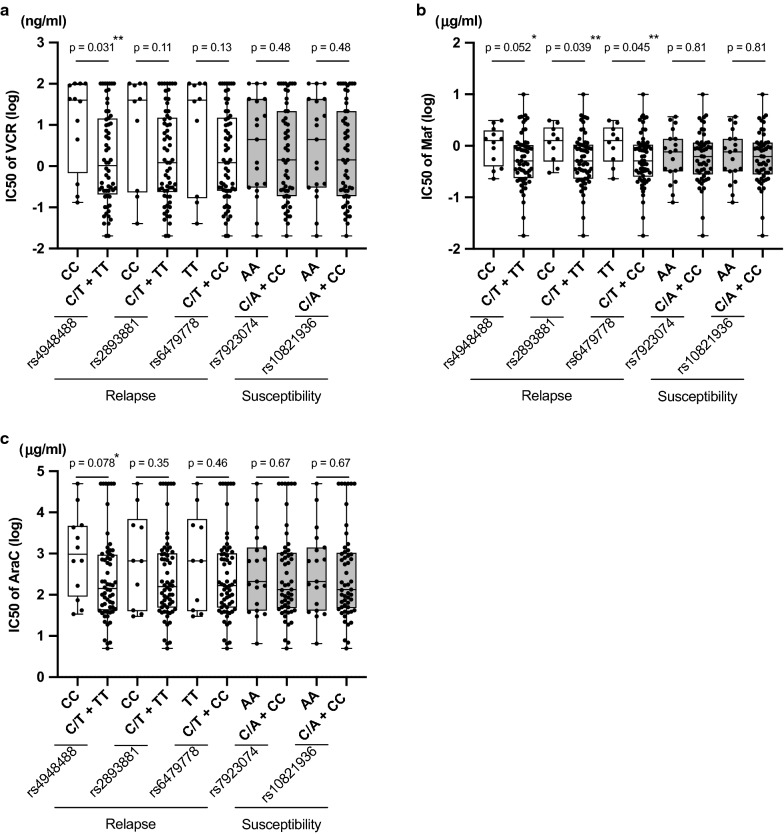
Association of relapse- and susceptibility-linked SNP genotypes with sensitivities to VCR (**a**), CY (**b**), and AraC (**c**). Vertical axis indicates log-scaled IC50 values of VCR (**a**), CY (Maf) (**b**), and AraC (**c**). The IC50 values of cell lines with homozygous genotype of risk allele and those with heterozygous or homozygous genotypes of non-risk allele in each SNP were compared. P-value in Mann–Whitney U test is indicated at the top of each SNP

Next we verified whether the genotypes of relapse-linked SNPs of *ARID5B* in BCP-ALL cell lines were associated with their sensitivities to chemotherapeutic agents. We performed an alamarBlue assay to determine IC50 values (concentration required to kill 50% of the cells) of nine representative chemotherapeutic agents [Pred, Dex, VCR, DNR, L-Asp, AraC, MTX, 6MP, and CY (Maf)] used for children with ALL. Of note, IC50 values of VCR (median IC50: 39.6 ng/ml) in 12 cell lines with homozygous risk allele (C) genotypes in the relapse-linked rs4948488 were significantly higher (p = 0.031 in Mann–Whitney U test) than those (1.04 ng/ml) in 60 cell lines with heterozygous or homozygous genotypes of the non-risk allele (T) (Fig. 3a). In addition to VCR, sensitivities to CY (Maf) (Fig. 3b) and AraC (Fig. 3c) tended to be associated with the genotype of the relapse-linked rs4948488. Similar associations were observed in genotypes of rs2893881 and rs6479778 (Fig. 3a–c). IC50 values of six agents (Dex, Pred, DNR, L-Asp, MTX, and 6MP) were not significantly associated with genotypes of the relapse-linked rs4948488, rs2893881, and rs6479778 (Additional file 3: Fig. S1a–f). Considering that *BCR/ABL1*-positive and *BCR/ABL1*-like ALL are characteristic entities, we analyzed the association in 56 BCP-ALL cell lines excluding 14 *BCR/ABL1*-positive and 2 *BCR/ABL1*-like ALL cell lines (Additional file 4: Fig. S2a–c). Similar associations were observed between genotypes of the relapse-linked SNPs of *ARID5B* and the sensitivities to VCR, CY (Maf), and Ara-C in *BCR/ABL1*-negative and *BCR/ABL1*-like-negative ALL cell lines.

We further analyzed any association of the susceptibility-linked rs7923074 and rs10821936 with drug sensitivities. In contrast to the genotypes of the relapse-linked SNPs, no significant associations were observed in genotypes of rs7923074 and rs10821936 with sensitivities to VCR, CY and AraC (Fig. 3a–c) and the other six chemotherapeutic agents (Additional file 3: Fig. S1a-f). These observations suggest that the risk allele of relapse-linked SNPs (but not susceptibility-linked SNPs) may be associated with a higher relapse rate in pediatric BCP-ALL patients due to reduced sensitivities to VCR, CY and AraC.

### Association of *ARID5B* gene expression with drug sensitivity

**Fig. 4:**
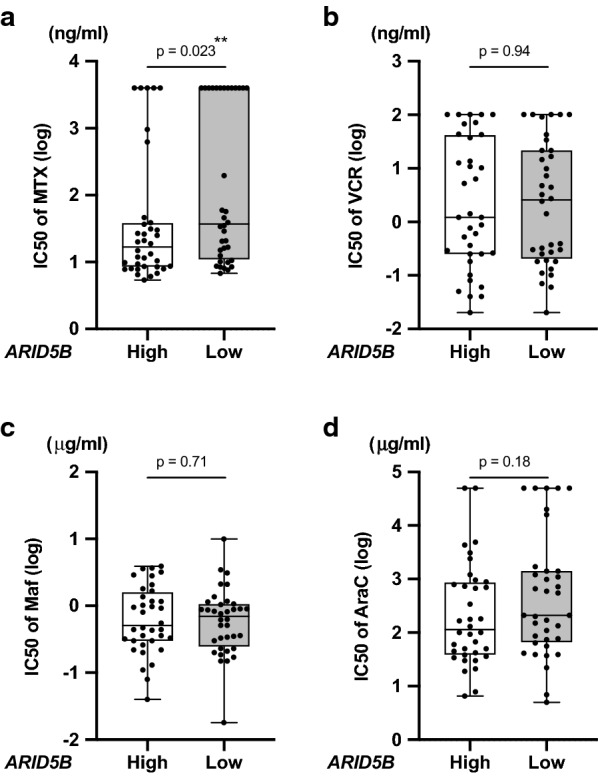
Association of *ARID5B* gene expression with sensitivities to MTX (**a**), VCR (**b**), CY (**c**), and AraC (**d**). Vertical axis indicates log-scaled IC50 values of MTX (**a**), VCR (**b**), CY (Maf) (**c**), and AraC (**d**). The IC50 values of 36 cell lines with higher *ARID5B* expression and the other 36 cell lines with lower expression were compared. P-value in Mann–Whitney U test is indicated at the top

Finally, we verified whether gene expression level of *ARID5B* was associated with drug sensitivities of BCP-ALL cell lines. To address this issue, we simply divided our 72 BCP-ALL cell lines into two groups—36 cell lines with higher than median value gene expression levels and 36 cell lines with lower than median value gene expression levels—and compared the IC50 values of each drug. Of note, the IC50 values of MTX in 36 cell lines with lower *ARID5B* expression (median IC50: 37.1 ng/ml) was significantly higher (p = 0.023 in Mann–Whitney U test) than those in the other 36 cell lines with lower expression (16.9 ng/ml) (Fig. 4a). A similar trend was observed in 56 BCP-ALL cell lines, excluding 14 BCR/ABL1-positive and 2 BCR/ABL1-like ALL cell lines (Additional file 5: Fig. S3). In contrast, although the sensitivities to VCR, CY, and AraC were associated with genotypes in the relapse-linked SNPs of *ARID5B*, no significant differences were observed in the IC50 values of VCR, CY, and AraC between the two groups (Fig. 4b–d). Furthermore, although genotypes in the susceptibility-linked SNPs of *ARID5B* were associated with sensitivities to Pred and Dex, no significant differences were observed in the IC50 values of Pred and Dex between the two groups (Additional file 6: Fig. S4a, b). In the IC50 values of the remaining three agents (DNR, L-Asp, and 6MP), there were no statistically significant differences between the two groups (Additional file 6: Fig. S4c-e). These observations suggest that lower *ARID5B* expression may be a genetic marker for MTX resistance in BCP-ALL.

### Association of *ARID5B* SNPs with cell cycle progression

Since anti-leukemic activity of chemotherapeutic agents are dependent on cell cycle progression, we analyzed the association of the susceptibility-linked and relapsed-linked SNPs of *ARID5B* with cell cycle progression in BCP-ALL cell lines. First, we compared cell cycle progression between cell lines with homozygous genotype of risk allele in each relapsed-linked SNP and those with the non-risk allele (Additional file 7: Fig. S5). No significant associations were observed in genotypes of the susceptibility-linked and relapsed-linked SNPs of *ARID5B* with cell cycle progression. Next, we compared cell cycle progression between the 36 cell lines with higher than median value *ARID5B* gene expression levels and the other 36 cell lines with lower than median value gene expression levels (Additional file 8: Fig. S6). No differences were observed between the two groups. These observations suggest that cell cycle progression may not directly be involved in the association of SNP genotype and gene expression level of *ARID5B* with drug sensitivities in BCP-ALL cell lines.

## Discussion

In the present study, using a series of BCP-ALL cell lines, we tried to verify the significance of genotype in the susceptibility-linked and relapsed-linked SNPs of *ARID5B* with *ARID5B* gene expression and drug sensitivities. It should be noted that the karyotypes in our cell lines were highly biased in comparison with those in childhood BCP-ALL patients: 14 cell lines were *BCR/ABL1*-positive, 13 cell lines were *TCF3/PBX1*-positive, 12 cell lines were *MLL* (*KMT2A*)-rearranged, and 3 cell lines were *TCF3/HLF*-positive. Furthermore, we later discovered that 15 cell lines were positive for *MEF2D*-fusions [18], which are recently identified fusion genes with a poor therapeutic outcome [19, 20]. In contrast, only four cell lines were *ETV6/RUNX1*-positive, and no cell lines were hyperdiploid. Thus, the majority of our cell lines were established from BCP-ALL with a poor prognosis.

Using these biased samples, we analyzed associations between genotypes of the relapsed-linked SNPs of *ARID5B* and the sensitivities to representative drugs for ALL treatment. We analyzed the sensitivities to nine agents, and found that sensitivities to VCR, CY, and AraC were associated with relapsed-linked SNPs; cell lines with homozygous genotypes of risk alleles in the relapsed-linked rs4948488, rs2893881, and rs6479778 were significantly more resistant to VCR, CY, and AraC than cell lines with non-risk alleles. These relapsed-linked SNPs of *ARID5B* were located in intron 2 of the *ARID5B* gene. However, no significant association was observed between genotypes of the relapse-linked SNPs of *ARID5B* and *ARID5B* gene expression level in BCP-ALL cell lines. Moreover, *ARID5B* gene expression level was not associated with sensitivities to VCR, CY, and AraC. Thus, genotypes of the relapse-linked SNPs of *ARID5B* are associated with VCR, CY, and AraC sensitivities of BCP-ALL cell lines independent of their *ARID5B* expression levels. Additionally, although cell cycle progression is highly associated with the drug sensitivity of leukemia cells, no clear association was observed between cell cycle progression and genotypes of the relapse-linked SNPs of *ARID5B* in BCP-ALL cell lines. Thus, further analyses are required to clarify the underlying mechanism behind the association between genotypes of the relapse-linked SNPs of *ARID5B* with the sensitivities to VCR, CY, and AraC in BCP-ALL cell lines.

Regarding the association of ARID5B with drug sensitivities in BCP-ALL cell lines, we also found that lower *ARID5B* gene expression level was associated with resistance to MTX. This finding seems to be partly consistent with a recent report by Xu et al. [15] who found that *ARID5B* knockdown in ALL cell lines led to specific resistance to MTX and 6MP. The authors showed that knockdown of *ARID5B* by using shRNA and CRISPR/Cas9 in ALL cell lines induced partial cell-cycle arrest at G0/G1 phase through upregulation of p21 [15], suggesting that cell-cycle arrest mediated by p21 may be involved in the induction of resistance to MTX and 6MP. However, in the present study, the proportions of G0/G1 phase in cell lines with lower *ARID5B* expression were similar to those in cell lines with higher *ARID5B* expression, suggesting that cell cycle progression was not a direct mediator in the association between *ARID5B* gene expression level and sensitivity to MTX in BCP-ALL cell lines. Further mechanism(s) other than cell cycle progression may be involved in the association of *ARID5B* expression with MTX sensitivity.

## Conclusion

In summary, our observations in 72 BCP-ALL cell lines suggest that the risk allele of the relapse-linked SNPs of *ARID5B* may be associated with higher relapse rates because of resistance to chemotherapeutic agents such as VCR, CY, and AraC. Moreover, lower *ARID5B* expression may be associated with MTX resistance. Limitations of the present study include that the study sample was restricted to limited numbers of leukemic cell lines with biased karyotypes and that underlying biological mechanisms for the associations remain unclarified. Since our findings were obtained from leukemia cell lines, further studies are needed before making a firm conclusion.

## Supplementary information

**Additional file 1: Table S1.** Karyotype, age, patient status at the cell establishment, and SNP genotypes and gene expression level of ARID5B in cell lines.

**Additional file 2: Table S2.** IC_50_ of each drug in cell lines.

**Additional file 3: Fig. S1.** Association of relapse- and susceptibility-linked SNP genotypes with sensitivities to Pred (a), Dex (b), DNR (c), L-Asp (d), MTX (e), and 6MP (f). Vertical axis indicates log-scaled IC50 values of Pred (a), Dex (b), DNR (c), L-Asp (d), MTX (e), and 6MP (f). The IC50 values of cell lines with homozygous genotype of risk allele and those with heterozygous or homozygous genotypes of non-risk allele in each SNP were compared. P-value in Mann–Whitney U test is indicated at the top of each SNP.

**Additional file 4: Fig. S2.** Association of relapse- and susceptibility-linked SNP genotypes with sensitivities to VCR (a), CY (Maf) (b), and AraC (c) in 56 BCP-ALL cell lines excluding 14 *BCR/ABL1*-positive and 2 *BCR/ABL1*-like ALL cell lines. Vertical axis indicates log-scaled IC50 values of VCR (a), CY (Maf) (b), and AraC (c). The IC50 values of cell lines with homozygous genotype of risk allele and those with heterozygous or homozygous genotypes of non-risk allele in each SNP were compared. P-value in Mann–Whitney U test is indicated at the top of each SNP

**Additional file 5: Fig. S3.** Association of *ARID5B* gene expression with sensitivities to MTX in 56 BCP-ALL cell lines excluding 14 *BCR/ABL1*-positive and 2 *BCR/ABL1*-like ALL cell lines. Vertical axis indicates log-scaled IC50 value of MTX. The IC50 values of 28 cell lines with higher than median value *ARID5B* expression veles and the other 28 cell lines with lower than median value expression levels were compared. P-value in Mann–Whitney U test is indicated at the top of each SNP

**Additional file 6: Fig. S4.** Association of *ARID5B* gene expression with sensitivities to Dex (a), Pred (b), DNR (c), LAsp (d), and 6MP (e). Vertical axis indicates log-scaled IC50 values of Dex (a), Pred (b), DNR (c), LAsp (d), and 6MP (e). The IC50 values of 36 cell lines with higher *ARID5B* expression and the other 36 cell lines with lower expression were compared. P-value in Mann–Whitney U test is indicated at the top

**Additional file 7: Fig. S5.** Association of relapse- and susceptibility-linked SNP genotypes with cell cycle progression. Percentage of G0/G1 phase was compared between cell lines with homozygous genotype of risk allele and those with heterozygous or homozygous genotypes of non-risk allele in each SNP. P-value in Mann–Whitney U test is indicated at the top of each SNP

**Additional file 8: Fig. S6.** Association of *ARID5B* gene expression with cell cycle progression. Percentages of G0/G1 phase in 36 cell lines with higher than median value *ARID5B* expression levels were compared with those in the other 36 cell lines with lower median value *ARID5B* expression levels. P-value in Mann–Whitney U test is indicated at the top

## Data Availability

The data used in present study are available from corresponding author on request.
