# Supplementary figures and images for: Association of relapse-linked ARID5B single nucleotide polymorphisms with drug resistance in B-cell precursor acute lymphoblastic leukemia cell lines
Source: Cancer Cell Int. 2020 Sep 4;20:434. doi: 10.1186/s12935-020-01524-0 (PMC7839197; doi:10.1186/s12935-020-01524-0)

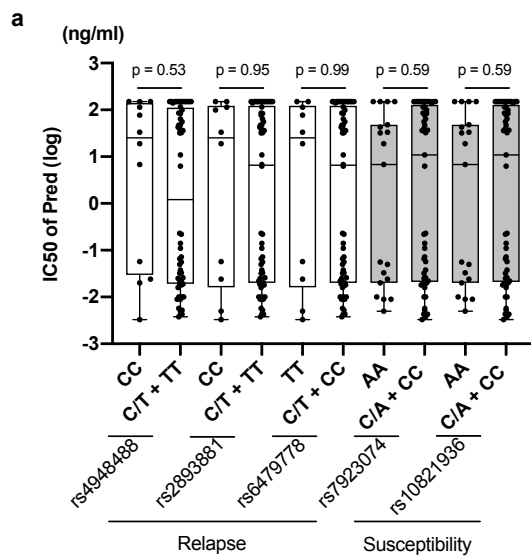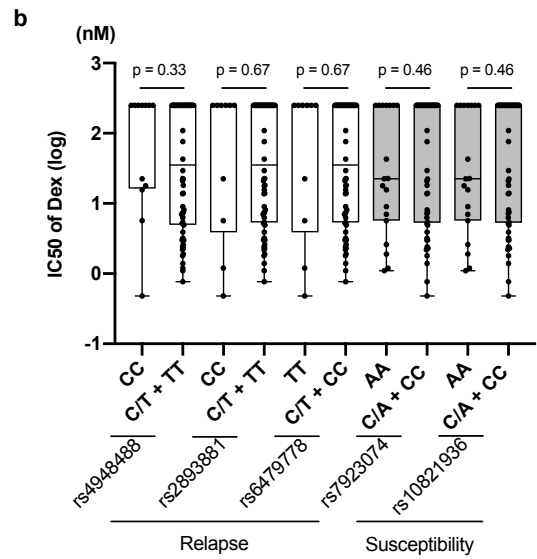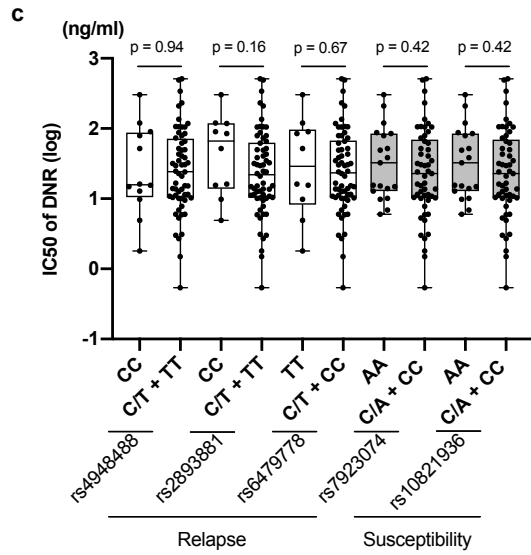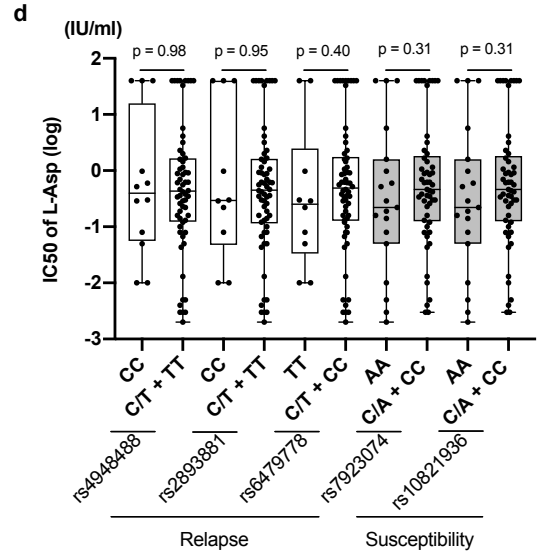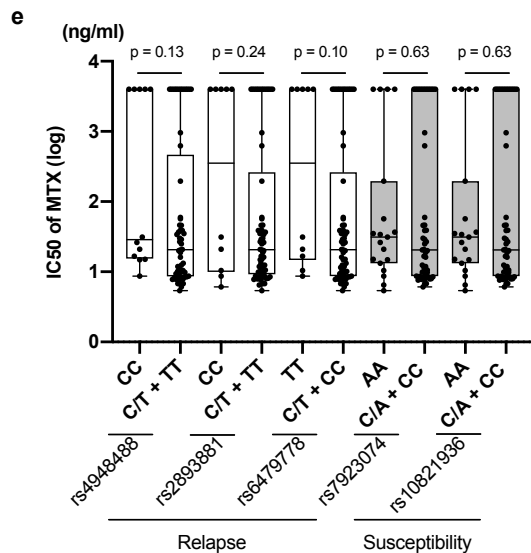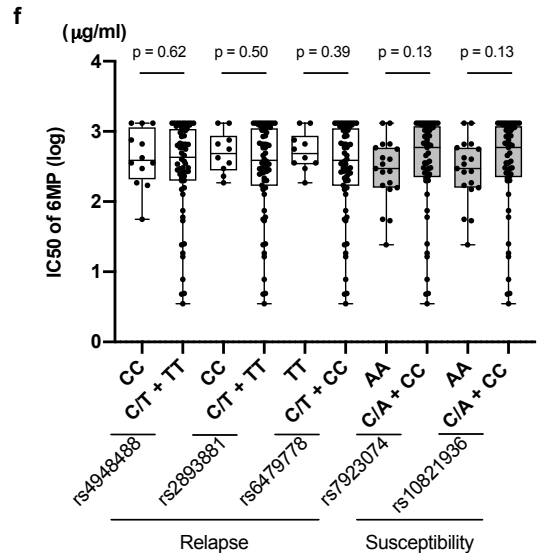

Supplement: Supplementary file 3 — Additional file 3: Fig. S1. Association of relapse- and susceptibility-linked SNP genotypes with sensitivities to Pred (a), Dex (b), DNR (c), L-Asp (d), MTX (e), and 6MP (f). Vertical axis indicates log-scaled IC50 values of Pred (a), Dex (b), DNR (c), L-Asp (d), MTX (e), and 6MP (f). The IC50 values of cell lines with homozygous genotype of risk allele and those with heterozygous or homozygous genotypes of non-risk allele in each SNP were compared. P-value in Mann–Whitney U test is indicated at the top of each SNP. [file 12935_2020_1524_MOESM3_ESM.pdf]

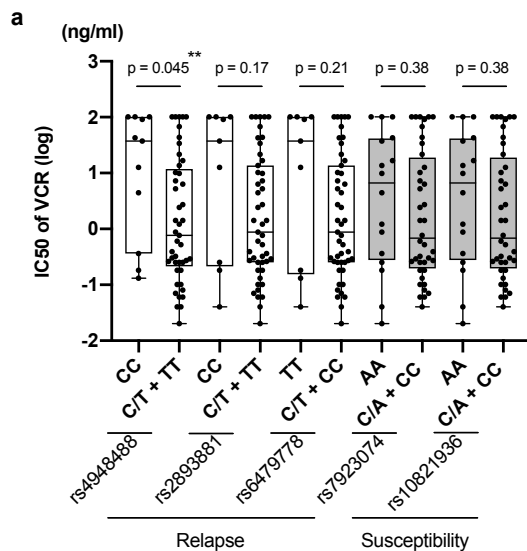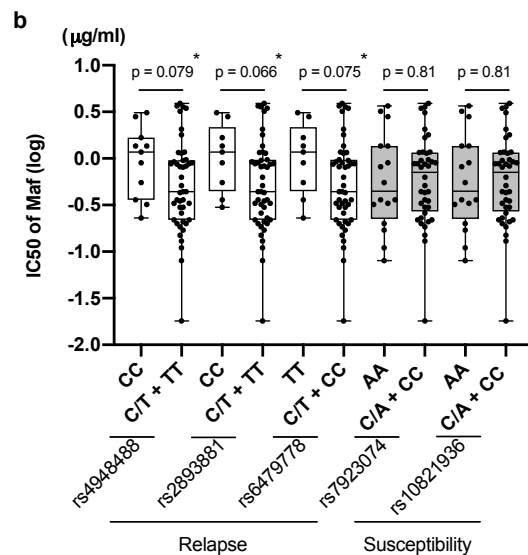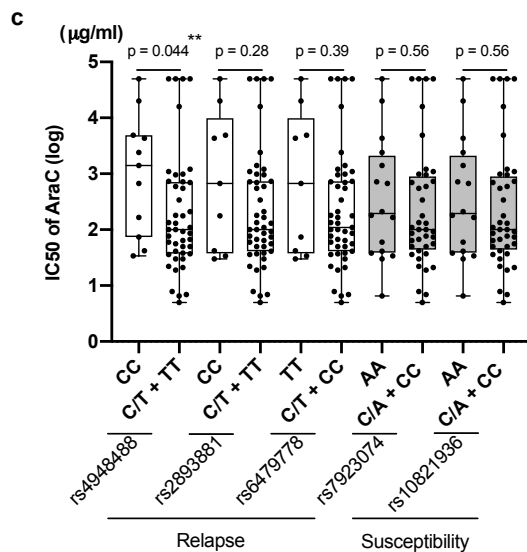

Supplement: Supplementary file 4 — Additional file 4: Fig. S2. Association of relapse- and susceptibility-linked SNP genotypes with sensitivities to VCR (a), CY (Maf) (b), and AraC (c) in 56 BCP-ALL cell lines excluding 14 BCR/ABL1-positive and 2 BCR/ABL1-like ALL cell lines. Vertical axis indicates log-scaled IC50 values of VCR (a), CY (Maf) (b), and AraC (c). The IC50 values of cell lines with homozygous genotype of risk allele and those with heterozygous or homozygous genotypes of non-risk allele in each SNP were compared. P-value in Mann–Whitney U test is indicated at the top of each SNP [file 12935_2020_1524_MOESM4_ESM.pdf]

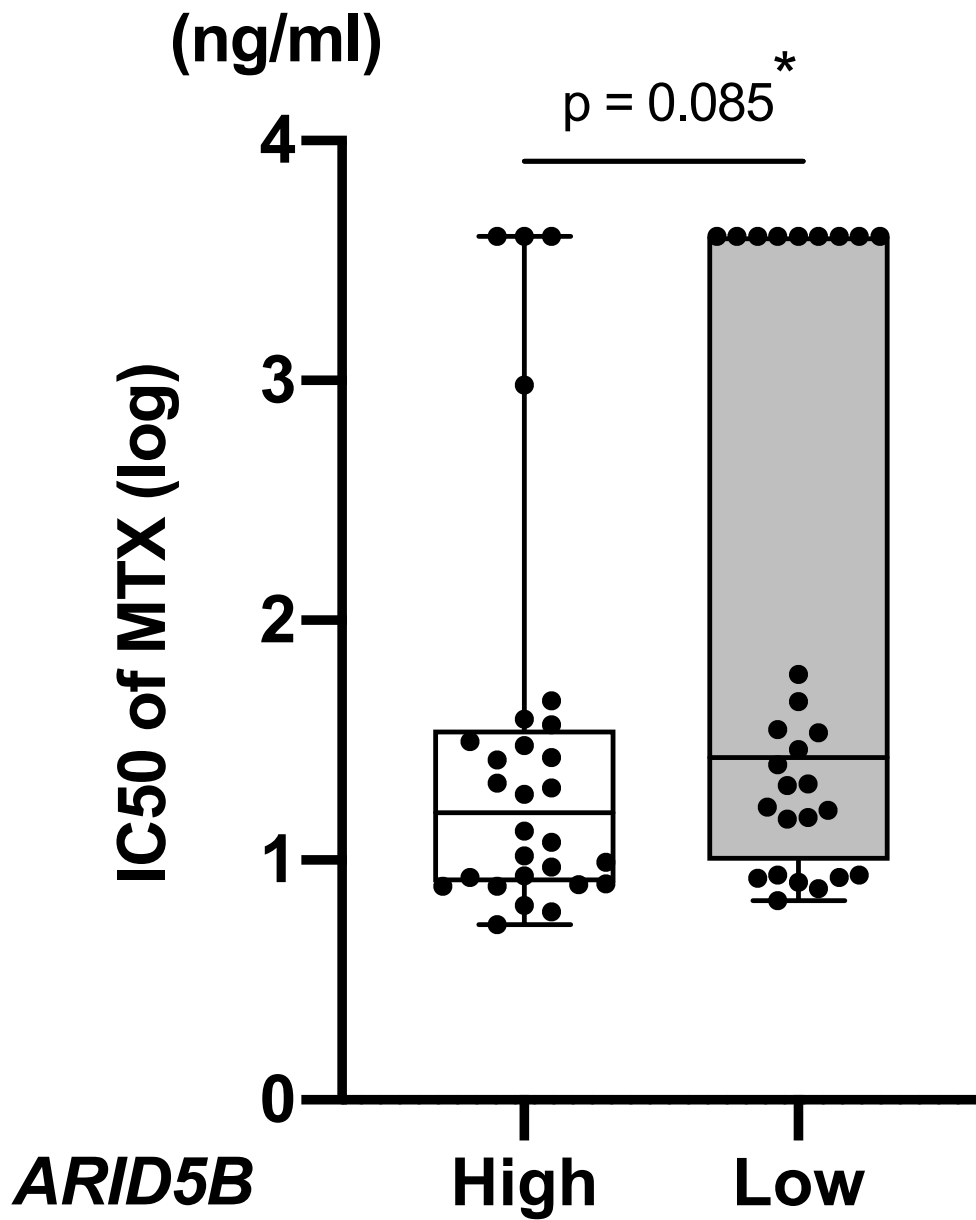

Supplement: Supplementary file 5 — Additional file 5: Fig. S3. Association of ARID5B gene expression with sensitivities to MTX in 56 BCP-ALL cell lines excluding 14 BCR/ABL1-positive and 2 BCR/ABL1-like ALL cell lines. Vertical axis indicates log-scaled IC50 value of MTX. The IC50 values of 28 cell lines with higher than median value ARID5B expression veles and the other 28 cell lines with lower than median value expression levels were compared. P-value in Mann–Whitney U test is indicated at the top of each SNP [file 12935_2020_1524_MOESM5_ESM.pdf]

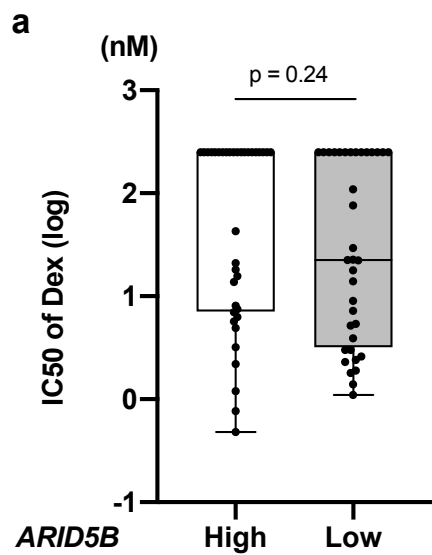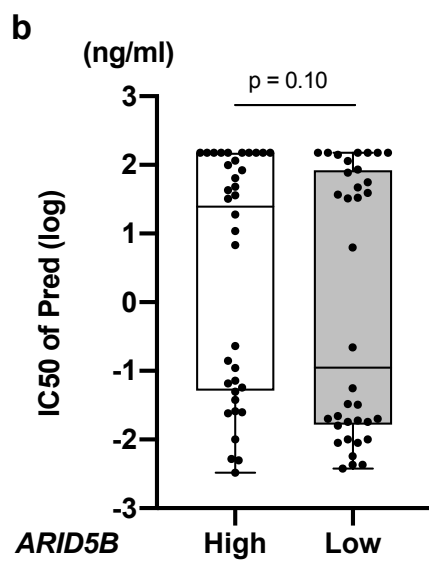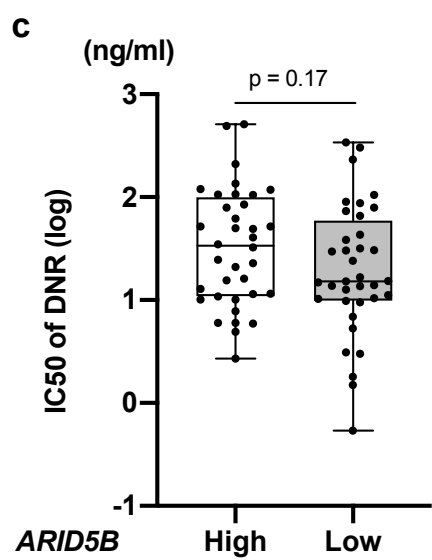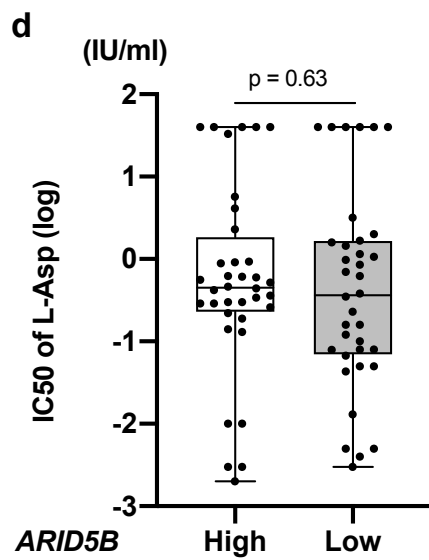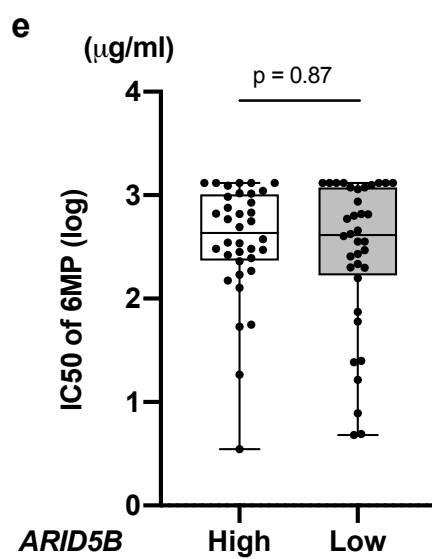

Supplement: Supplementary file 6 — Additional file 6: Fig. S4. Association of ARID5B gene expression with sensitivities to Dex (a), Pred (b), DNR (c), LAsp (d), and 6MP (e). Vertical axis indicates log-scaled IC50 values of Dex (a), Pred (b), DNR (c), LAsp (d), and 6MP (e). The IC50 values of 36 cell lines with higher ARID5B expression and the other 36 cell lines with lower expression were compared. P-value in Mann–Whitney U test is indicated at the top [file 12935_2020_1524_MOESM6_ESM.pdf]

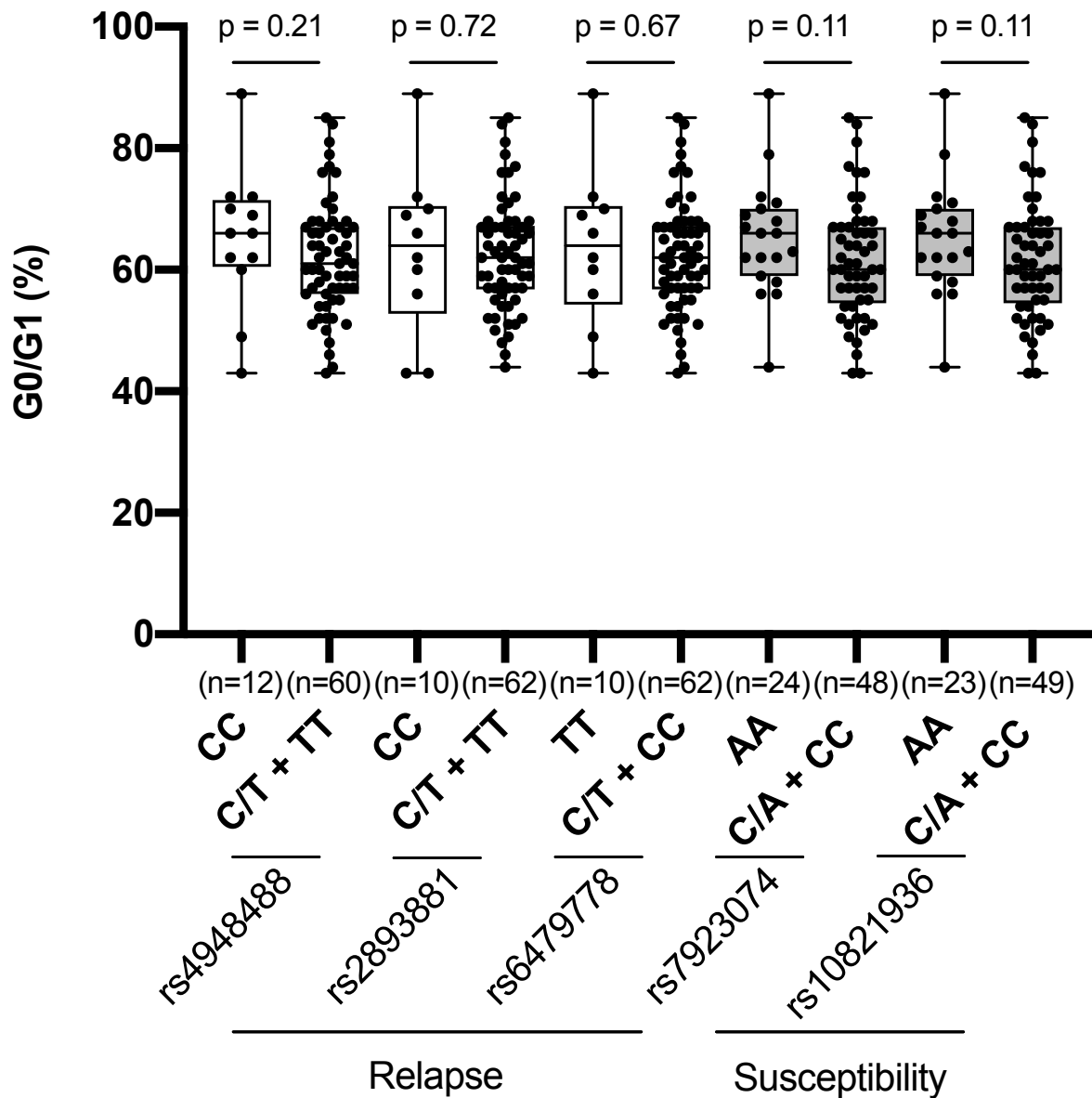

Supplement: Supplementary file 7 — Additional file 7: Fig. S5. Association of relapse- and susceptibility-linked SNP genotypes with cell cycle progression. Percentage of G0/G1 phase was compared between cell lines with homozygous genotype of risk allele and those with heterozygous or homozygous genotypes of non-risk allele in each SNP. P-value in Mann–Whitney U test is indicated at the top of each SNP [file 12935_2020_1524_MOESM7_ESM.pdf]

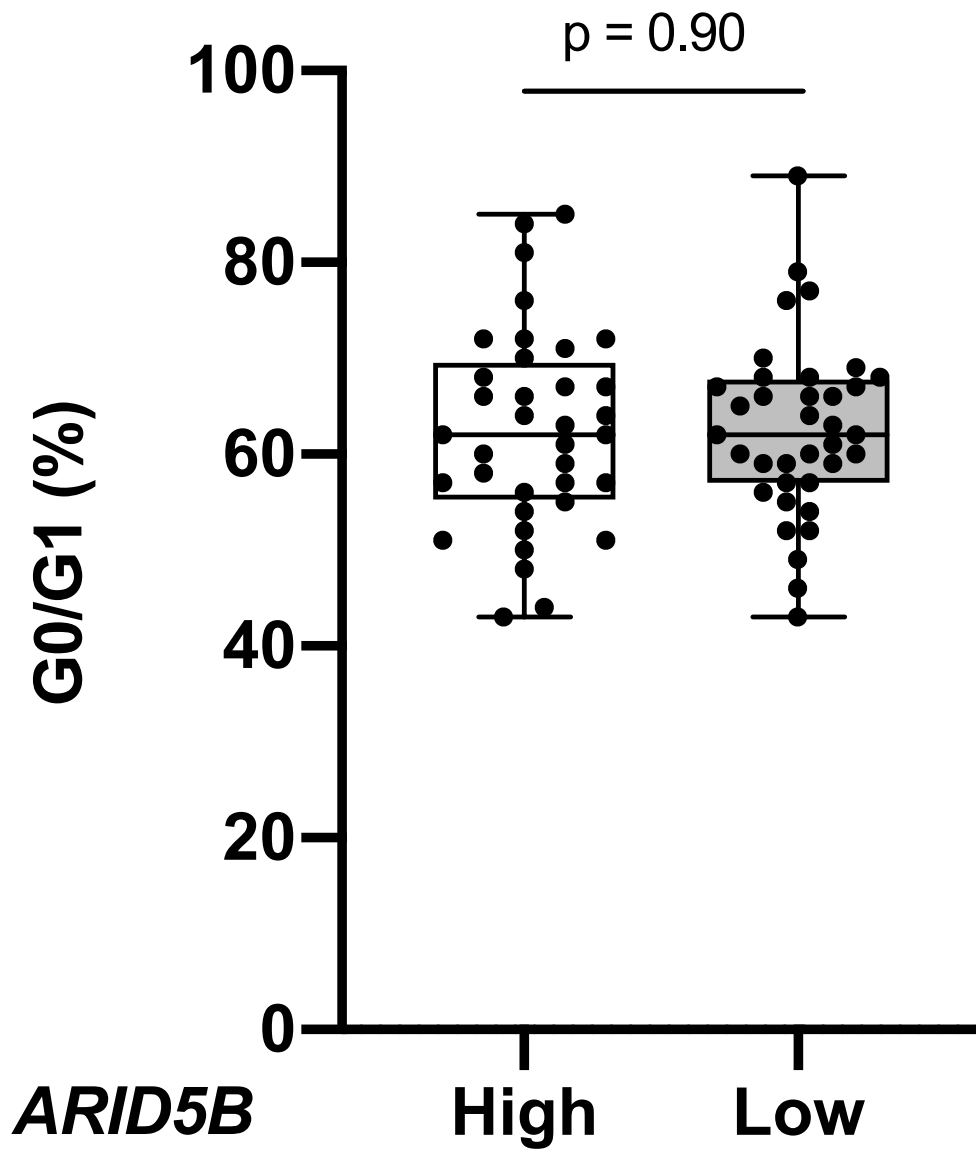

Supplement: Supplementary file 8 — Additional file 8: Fig. S6. Association of ARID5B gene expression with cell cycle progression. Percentages of G0/G1 phase in 36 cell lines with higher than median value ARID5B expression levels were compared with those in the other 36 cell lines with lower median value ARID5B expression levels. P-value in Mann–Whitney U test is indicated at the top [file 12935_2020_1524_MOESM8_ESM.pdf]
